# Epidemiological Analysis of Childhood Brucellosis in Duhok, Iraq: A Retrospective Observational Study From 2018 to 2021

**DOI:** 10.7759/cureus.64934

**Published:** 2024-07-19

**Authors:** Teroj Mohamed, Nizar Bakir Yahya

**Affiliations:** 1 Dental Basic Sciences, University of Duhok, Duhok, IRQ; 2 General Medicine and Surgery, College of Medicine, University of Duhok, Duhok, IRQ; 3 Pediatrics, Heevi Pediatrics Teaching Hospital, Duhok, IRQ

**Keywords:** public health intervention, zoonosis and public health, pediatric infections, epidemiology, brucellosis

## Abstract

Brucellosis is a zoonotic disease that can spread from animal to human through contaminated dairy products, raw vegetables, and water or via direct contact with infected animal tissues. While the disease affects individuals of all ages, research on its prevalence and clinical profile, particularly in Iraq, is limited. To address this knowledge gap, a retrospective observational study was conducted using data from the Bacterial Serology Laboratory of Heevi Pediatric Teaching Hospital, Duhok, Iraq, from 2018 to 2021. Of the 1,032 suspected cases of brucellosis in children, 72 cases that met the inclusion were analyzed. The study found a 7% prevalence of childhood brucellosis in the Duhok region, with the highest incidence of 7.8% in 2020. The infection rate was age-dependent, with most cases occurring in the year’s third quarter. No statistically significant relationship was found between sex and the infection rate. These findings highlight the importance of targeted public health interventions and improved diagnostic protocols to reduce the prevalence of brucellosis among children in this region.

## Introduction

Brucellosis, a zoonotic disease caused by the gram-negative, facultative intracellular bacteria *Brucella* spp., poses a significant threat to global human health and livestock productivity, particularly in low-income and developing countries [1]. While primarily affecting animals, brucellosis can be transmitted to humans through contact with infected animals and consuming contaminated animal products. Individuals working near livestock, such as those in agricultural settings or abattoirs, are at heightened risk [2].

The clinical presentation of *Brucella* infection is often nonspecific and can impersonate other infectious and inflammatory conditions, making diagnosis challenging [3]. Symptoms range from fever, sweats, and musculoskeletal pain to potentially life-threatening complications, such as endocarditis and neurobrucellosis. Accurate and timely diagnosis is crucial to guide appropriate treatment and prevent long-term sequelae [4-6].

Brucellosis affects people of all ages, including infants and children [7]. There is limited research on the prevalence and clinical profile of childhood brucellosis, particularly in Iraq. To address this knowledge gap, we retrospectively reviewed suspected cases of childhood brucellosis among pediatric patients attending Heevi Pediatric Teaching Hospital in the Duhok Governorate. The study aimed to achieve four main objectives: to estimate the prevalence of childhood brucellosis, to detail the clinical profiles and laboratory findings in affected children, to analyze the seasonal variation in brucellosis cases among the pediatric population, and to find any change in number of cases after the COVID-19 pandemic.

## Materials and methods

Study design

This cross-sectional, retrospective observational study investigated childhood brucellosis’s prevalence and clinical profile between January 2018 and December 2021.

Data source

Data were sourced from the Bacterial and Serological Laboratories of Heevi Pediatric Teaching Hospital in the Kurdistan region of Iraq. This hospital specializes in pediatric care and handles samples from various regional districts. Diagnostic data from 1,032 suspected cases of *Brucella* infection in children was collected and sorted using Microsoft Excel 2016.

Study area

**Figure 1:**
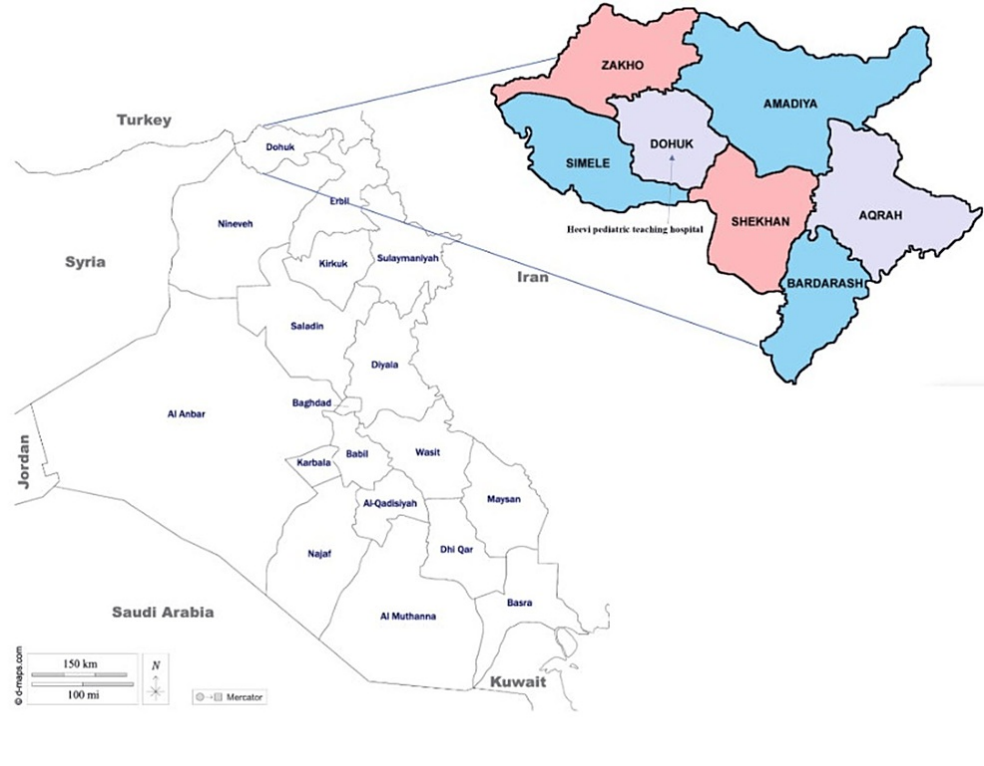
Map of the Duhok Governorate with the six surrounding districts and reference to the Heevi Pediatric Teaching Hospital

Duhok, the capital of the Duhok Governorate, is located in the northwest Iraqi Kurdistan region. This city has a population of approximately 2.3 million, including host community members, internally displaced people, and refugees [8]. The climate is semi-arid, with hot summers and cool winters [9]. Heevi Pediatric Teaching Hospital is the region’s only specialized children’s hospital [10] (Figure 1).

Study population

The study included children aged one day to 18 years who tested positive for the Rose Bengal test. Patients undergoing treatment for brucellosis were excluded to ensure that the study population consisted solely of newly diagnosed cases.

Inclusion and exclusion criteria

**Figure 2:**
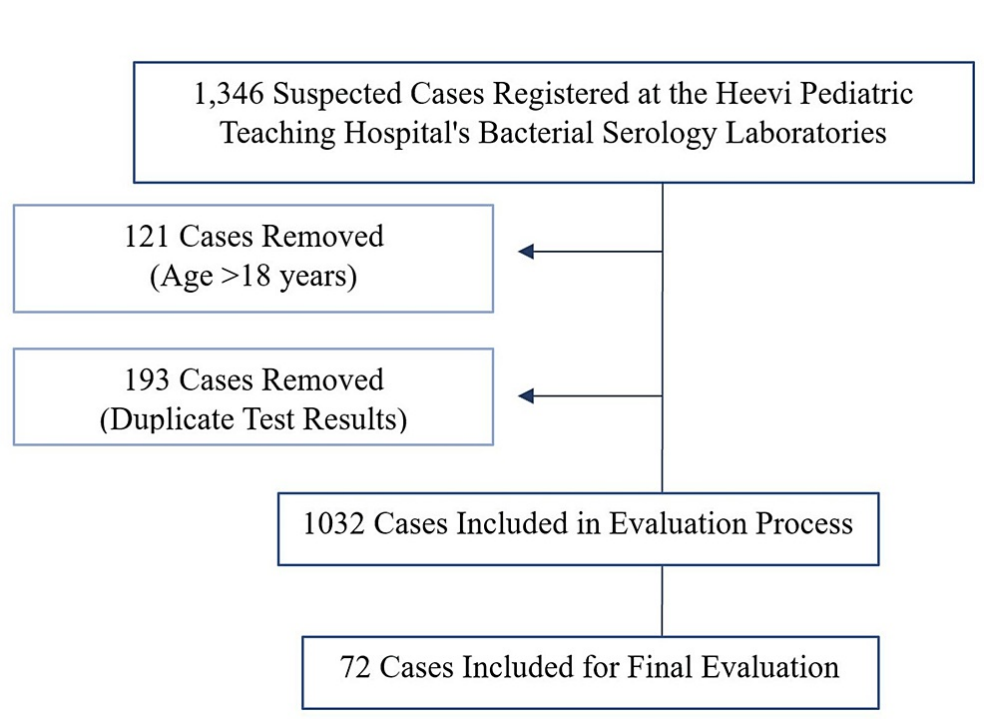
Flow chart of the inclusion and exclusion criteria for suspected brucellosis cases

Out of the initial screening of 1,346 suspected cases, 1,032 were included based on the initial inclusion criteria. Seventy-two tested positive for brucellosis using the Rose Bengal test and met all the inclusion criteria for the final evaluation. The remaining cases tested negative and were excluded. The process of selecting these cases is illustrated in the flow chart (Figure 2).

Statistical analysis

Descriptive analysis was based on the frequency of *Brucella* sample seropositivity, stratified by sex, age, and year of testing. Using the chi-square test, inferential statistical analysis was used to test the association between brucellosis cases and season. The Kruskal-Wallis test identified significant differences between normal and abnormal laboratory findings, with statistical significance set at a p-value of <0.05. Numerical variables are expressed as mean ± standard deviation and categorical variables as numbers (percentages). The data were analyzed using IBM SPSS Statistics, version 25.0 (IBM Corp., Armonk, NY).

## Results

Demographic findings

**Figure 3:**
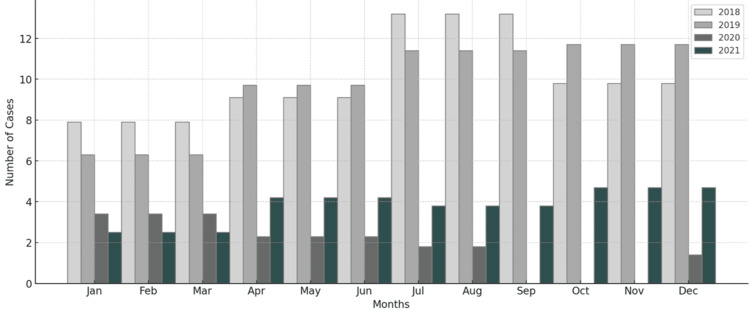
Annual distribution of suspected brucellosis cases from 2018 to 2021 at Heevi Pediatric Teaching Hospital The total number of serum samples tested for *Brucella* antibodies decreased substantially after March 2020, coinciding with the first wave of the COVID-19 pandemic.

Of 1,032 serum samples tested from 2018 to 2021, 554 (53.7%) were from males and 478 (46.3%) from females. The median age of the suspected patients was seven years, with a standard deviation (SD) of 4.3 years (95% CI, 7.45-7.98 years). The total number of children tested for *Brucella* antibody detection decreased dramatically after March 2020, coinciding with the first wave of the COVID-19 pandemic (Figure 3).

Prevalence of childhood brucellosis

Seventy-two cases were confirmed positive for brucellosis, resulting in a prevalence of 7% among the tested pediatric patients at Heevi Pediatric Teaching Hospital over the four years. The highest prevalence of infected children (7.8%) was observed in 2020 (seven out of 89 serum samples tested). The median age of the children infected with *Brucella* spp. was nine years (IQR, 7.75-10.3; 95% CI, 8.3-10.3). A significant relationship was observed between age and infection rate (X2 = 16.67 when df = 5, N = 1032, p < 0.001). Of the infected children, 38 (52.8%) were male and 34 (47.2%) were female, but no statistically significant relationship was observed between sex and infection rate. 

Seasonal distribution

**Figure 4:**
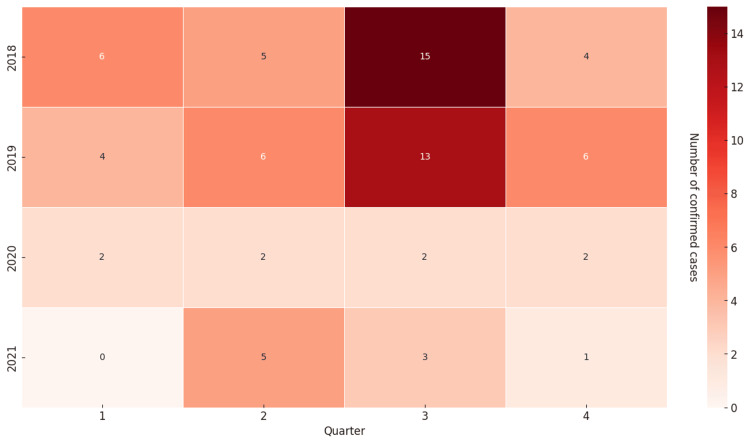
Seasonal distribution of brucellosis cases from 2018 to 2021 The highest incidence of brucellosis was observed during the third quarter of the year, corresponding to summer and early autumn.

Most cases were detected in the third quarter of the year, specifically from late June to early October, which corresponds to summer and early autumn. This finding indicates a statistically significant relationship between the season and the peak number of cases, with a higher incidence observed during summer (Figure 4).

Impact of COVID-19

A comparison of the number of suspected cases before and after the COVID-19 pandemic showed a significant decrease. Specifically, the number of suspected cases sharply decreased from 22.9 to 10 per month between 2020 and 2021 following the onset of the COVID-19 pandemic. The relationship between diagnostic test results and year, as well as year and season, remains essentially unchanged.

Laboratory findings

The laboratory records of 44 children showed abnormal blood parameters, with 84.09% showing elevated erythrocyte sedimentation rate (ESR). Hemoglobin (Hb), white blood cell (WBC) count, platelet count, and lymphocyte count were frequently below average (Table 1).

**Table 1:** Hematological findings in children suspected of brucellosis This table shows the frequency and mean values of abnormal blood parameters in a sample of 44 children diagnosed with brucellosis through the Rose Bengal test. HB, hemoglobin; WBC, white blood cell count; ESR, erythrocyte sedimentation rate

Clinical finding	Number of patients (%)	Mean ± SD	p-value
HB (g/dL)	27 (61.36%)	10.95 ± 1.578	0.692
WBC (×10^3^ cells/μL)	11 (25%)	7.21 ± 3.574	0.4
Lymphocyte count (×10^3^ cells/μL)	23 (52.27%)	3.25 ± 1.571	0.1
Platelet count (×10^3^ cells/μL)	8 (18.18%)	270.66 ± 124.827	0.51
ESR (mm/h)	37 (84.09%)	28.18 ± 18.489	0.278

## Discussion

Childhood brucellosis remains a major public health concern in many developing countries and endemic areas worldwide, including the Duhok Governorate in the Kurdistan region of Iraq [11,12]. Brucellosis is frequently associated with occupational exposure or the consumption of unpasteurized dairy products in this region. Our study revealed a 7% prevalence of *Brucella* spp. infection among suspected pediatric patients at Heevi Pediatric Teaching Hospital from 2018 to 2021. This rate is higher than the 1.6% prevalence reported in a 2004 study published in the Journal of Tropical Pediatrics, which assessed blood samples over a 13-year period [13]. Prevalence rates vary depending on the study population, geographical location, diagnostic methods, and endemic *Brucella* species. Our study’s high prevalence rate of childhood brucellosis may be attributed to exposure to occupational, environmental, or dietary sources of pathogens. Consuming unpasteurized dairy products is a significant risk factor for infection. However, infection in patients younger than one year may be underreported or misdiagnosed due to a less severe course of illness and nonspecific symptoms in infants.

Several studies have explored the relationship between childhood brucellosis and sex, but the results have been inconsistent. Our study found no significant difference in the incidence of brucellosis between males and females, consistent with results from studies in Turkey and Skopje [13,14]. In contrast, studies from West Iran, Jordan, and Saudi Arabia reported a higher infection rate in males [15-18], possibly due to the direct involvement of young boys in livestock farming, which increases the risk of contracting the disease.

The epidemiological prevalence of *Brucella* infection is complex and influenced by factors such as animal disease prevalence, human behavior, and environmental conditions. Studies in countries like Iran, Turkey, and Iraq have examined the seasonal pattern of *Brucella* infection, reporting higher rates during the summer and autumn [12,14,15,17,19]. This seasonal variation could be attributed to the increased activity and movement of livestock and the higher temperature, which can favor the growth and transmission of bacteria [20]. Additionally, breeding and slaughtering animals during summer could contribute to the observed seasonal variation. However, studies conducted in Iran, Georgia, Taiwan, and China found a non-clear relationship between the seasonal distribution and reported cases throughout the year [21-23].

Our study observed a significant decrease in the number of suspected cases from 2019 to 2021, which several factors may have influenced. These include increased public awareness, changes in agricultural practices, animal husbandry, or modifications to testing policies. Additionally, during the COVID-19 pandemic, many healthcare resources were redirected to managing the pandemic, potentially leaving fewer resources available for testing and treating other diseases. A study conducted in Iran supported our findings, reporting a decrease in cases and changes in the epidemiology and pattern of *Brucella* infection [24].

Brucellosis is a systemic disease, with *Brucella* bacteria capable of invading any organ, causing symptoms such as fever, myalgia, and arthralgia of the major joints. Laboratory findings in patients with brucellosis often include anemia, leukopenia, thrombocytopenia, elevated liver enzymes, increased CRP, and increased ESR. The elevated ESR in 37 patients indicated inflammation and/or disease activity, and the ESR can serve as a marker for *Brucella* infection when other characteristic symptoms are present. Anemia in children with brucellosis may result from bone marrow suppression and chronic infection, detected in 27 (61.36%) patients. Additionally, leukocytosis, lymphocytosis, and thrombocytopenia were increased in 11 (25%), 23 (52.27%), and 8 (18.18%) of the patients, respectively. These hematological abnormalities can be greater than previously reported [3,5,25,26], and low platelet counts may predispose patients to bleeding complications [27].

Study limitations 

This study has several limitations. Its retrospective design and single-center setting may limit the generalizability of findings. The relatively small sample size (72 confirmed cases of 1,032 suspected cases) may have affected statistical power and introduced selection bias. The impact of the COVID-19 pandemic on healthcare access and testing practices may have influenced the observed trends. Additionally, the study did not comprehensively account for all potential environmental and agricultural factors, nor did it include detailed clinical outcomes and long-term follow-up data. The reliance on the Rose Bengal test, with its inherent variability in sensitivity and specificity, is another limitation. Future research should address these limitations through perspective designs, larger multi-center studies, and the inclusion of comprehensive clinical data.

## Conclusions

This study illuminates the persistent public health concern of childhood brucellosis in the Duhok Governorate. Our findings reveal a distinct seasonal pattern, with cases peaking during summer, suggesting a potential association with environmental or agricultural factors. While age emerged as an influencing factor in infection rates, no significant gender disparities were observed. Furthermore, the COVID-19 pandemic demonstrably impacted the number of suspected cases. These insights underscore the critical need for sustained public health interventions to mitigate childhood brucellosis in the region. Prioritizing preventative measures and implementing comprehensive community education programs are essential steps toward safeguarding children’s health.
